# The problem of waiting lines for otorhinolaryngology surgeries in public services

**DOI:** 10.1016/S1808-8694(15)31321-5

**Published:** 2015-10-20

**Authors:** Krishnamurti Matos de Araujo Sarmento, Shiro Tomita, Arthur Octavio de Avila Kos

## INTRODUCTION

According to Article 196 of the Brazilian Constitution “Health is a right of all citizens and a duty of the State”. The constitution is supplemented by the organic Act 8080, dated from 1990, which sets the guidelines for the Brazilian universal health service, SUS (in Portuguese Sistema Único de Saúde), ensuring universal access to health services.

In spite of the legislation's theoretical excellence, it is common knowledge that access to public services is one of the most serious problems in our society. The factors leading to this scenario are, unquestionably, absence of an efficient and hierarchical structure, scarce financial resources for health and not enough investments in hospitals, health professionals and technology.

Waiting lines are lists of patients requiring the same treatment or medical services whose demand is larger than the supply. Metaphorically speaking, patients in this line are kept in a virtual room, waiting for the same procedure, and are called on a one-by-one basis, according to order of arrival. The waiting line for elective surgery is a reality in many general hospitals throughout Brazil, which can be longer or shorter in terms of number of patients and time in wait.

Although an integral part of the daily routine of surgeons working in public service facilities, the waiting line issue is not frequently tackled by the medical-scientific community, owing perhaps to the fact that this seems to be a discussion that does not belong in the academic sphere and that should be circumscribed to governmental instances. However, it must be highlighted that equitable, fair and universal access to health services must be a constant concern not only of governments but also of all professionals involved with the public service network. There is a lot that can be done locally to mitigate waiting lines.

This article tries to discuss this issue from our specialty's perspective, bringing up some points that we consider to be of great relevance.

### Waiting lines for surgery: The tip of the iceberg

**Figure 1:**
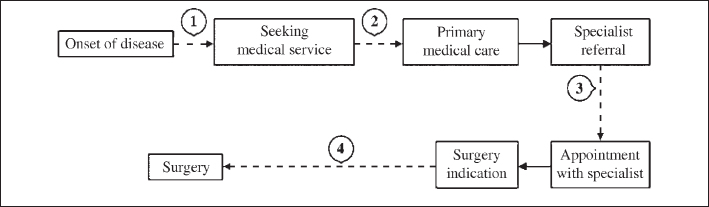
Arrows represent critical points in attempting to get treatment.

Before being submitted to otorhinolaryngologic surgeries in the public network, patients must actually sit on several consecutive waiting lists. The waiting time for surgery —representing the time between surgical indication and its actual performance— is but the last, and many times the shortest, of these waiting lines. Total real wait-time covers many other previous time-spans, starting at symptom onset and ending when specialized treatment is finally delivered. Each one of the steps is characterized by its own difficulties and delays (Figure 1). The phases prior to treatment at the otorhinolaryngology service are much more difficult to size, but are not less important. All different phases are worthy of special and knowledgeable attention so that obstacles may be identified, feasible solutions may be found and the flow of patients may be optimized.

First of all, one can see that there is a time lag between the onset of the disease and the action of seeking medical help (arrow 1 in Figure 1). This is partly due to lack of information on otorhinolaryngologic diseases and the most important presentations requiring medical attention. Information campaigns on Voice and Hearing, such as the ones supported by the Brazilian Society of Otorhinolaryngology are instrumental for changing this scenario.

Next we have the difficulty in obtaining medical services (arrow 2), a consequence of the problems in our health policy, its faulty structure and the lack of resources for health care in Brazil.

### Getting an appointment with a specialist: difficulties and distortions

The crucial point for these patients is, perhaps, getting an appointment at the otorhinolaryngology outpatient clinic (arrow 3). Everybody knows that public service wards are overcrowded. In many cases, it takes many months or, sometimes, more than a year to get an appointment at these facilities, generating pent-up demands of enormous proportions. The referral/counter-referral system —supposedly to provide for a specialist “opinion” and to establish a hierarchy of services—, has not been satisfactorily implemented yet. Again, the lack of human and logistic resources is appalling.

These difficulties which, by and large, require government action, generate several medical service distortions that may be identified by the otorhinolaryngologist and that must be corrected at the local level, with great impact to promoting the population's health. One of such required measures has to do with extra appointments.

Faced with the impossibility of making an appointment following regular procedures, it is only natural who can resort to other means of getting to see the doctor will do so. This creates a demand for “extra appointments”, the so-called “EFF requests” or “employee-family-or-friend request”.

The number of these multiplies at great speed. Pressure is exerted not only upon the otorhinolaryngologist himself, but also on all those who have access to him: nurses, supporting staff, physicians from other specialties etc. The employees who are in charge of making these appointments are also subject to pressure; one must pay heed to the fact that even those patients who seem to have made their appointments via regular means may have gotten there through special favors. Even more serious is the practice (i.e. crime) that some employees engage in; selling appointments or selling places at the hospital's screening line. The doctor becomes, unbeknownst to him, a gear in a profitable business: i.e. brokerage of public medicine.

In our specialty, this problem is complicated even further by the fact that people usually expect our visits to be “quick”, and our problems simple to solve. Patients will more readily accept “extra-service” refusals from a cardiology clinic, understanding that the doctor cannot just “take a quick glance at his heart”, than accept the same type of refusal by an otorhinolaryngologist. Everybody knows that anamnesis and physical examination of patients with heart diseases take time and frequently require other examinations. On the other hand, an otorhinolaryngologist's refusal to “take a quick look” at one's ear, or just prescribe a little something for sinusitis sounds like unwillingness to accommodate a simple request.

It is true that most presentations in otorhinolaryngology are quick to diagnose and treat, not even requiring a second visit. However, there are many instances where longer examinations are required, with repeated visits, supplementary audiologic tests, endoscopy and, ultimately, surgery. One cannot deny that these patients contribute to the over-crowding of our outpatient clinics and are an important factor adding to the difficulty other patients have in obtaining an appointment with our specialists.

In order to tackle the problem of extra appointments one must, first of all, make it visible. “Extra” patients —whatever the severity of their disease may be, and acknowledging their undeniable right to medical service— are actually “line-jumping” i.e. passing ahead of other people waiting in a virtual line. This can lead to extreme scenarios where the second line flows continually and the main line –in other words, the legitimate one— remains practically still. The number of patients submitted to surgery who were somewhat favored on their initial appointment at the otorhinolaryngology service is surprising. But one can only see the true picture if extra appointments are accounted for. Thus, it is absolutely necessary that a recording mechanism be created to register such appointments. This will also make the specialist work look more valuable; otherwise the time spent with these patients is classified as idle.

The so-called “corridor visits” should also be avoided. If one chooses to provide medical assistance to an extra patient, this service should be provided in full. Otherwise we will be corroborating the idea that a visit to an otorhinolaryngologist is tantamount to a “quick glance”— diminishing and making our work look vulgar. Extra patients should preferably be seen after the last regularly-scheduled patient.

### Short line X Long line

A short line of people awaiting surgery is not necessarily synonymous with more efficient medical services. Many medical services choose to maintain short lines by resorting to artificial tricks; i.e. refusing to accept, for a period of time, new patients with diseases that demand surgery, or only accepting new patients as the ones that are already in line are operated on. This makes for a constant low number of *registered* patients waiting for surgery.

A superficial analysis might lead us to think that this is a plausible solution from the hospital administrative perspective, making it easier to manage and provide services to patients. It might also be argued that by denying medical service to these patients one is actually forcing them to look elsewhere for medical treatment and that if other institutions did the same this would ultimately lead to the civil society putting pressure on the government authorities demanding a solution to the problem.

A more careful examination of the issue, however, shows that the “short-line” expedient must be avoided. First of all because this attitude has a negative impact upon the lives of those who were denied service –which is against medical ethical principles. Additionally, this practice creates a false sensation of bringing the problem under control, and drifts health professionals away from reality.

The thesis advocating denial of service as the only way to force a patient to fight for his rights is absurd and anti-ethical. Patients have a right to being informed about what is happening as much as they have a right to health, and no one has the right to treat patients as a “device” that should take this or that attitude, even if we believe that this is to the patient's benefit. Besides, civil society pressure would hardly emerge among patients deprived of information and resources and, more importantly, without the participation of specialists directly involved —and who, with this type of attitude, would be waiving their responsibility to act. Trying to disguise service refusal to these patients as a political attitude that would ultimately revert to their benefit is naïve or, more frequently than not, a defense mechanism to mitigate their anxiety.

Even though one may argue that it is not fair to accept patients for treatment when we know we cannot provide surgical services, one must pay heed to the fact that surgery is but one of the health-promoting measures – albeit sometimes the most important one. Even though a specific condition may have solid indication for surgery, one cannot deny the benefit and the positive impact on the patient's quality of life that is obtained from any medical attention provided in a dignifying manner –including clarifications about the disease and its possible complications, its natural history and clinical treatment, palliative as it may be. The fact that we cannot treat the disease in the manner that seems ideal to us does not waive us from the responsibility of treating the patient as best we can.

If we are to engage in a serious policy to challenge this issue, in addition to providing patients newly admitted to the waiting lines with explanations about their condition, we should inform them, preferably in writing, about their surgical indication (explaining the reasons why this is the best course of action), tell them that there is a waiting line and explain the prioritization criteria, the number of people waiting and the expected waiting time for surgery — highlighting that this is just an estimate. We should also make clear that it would be preferable that the surgery be made as soon as possible, enumerate possible complications and sequelae that may ensue from waiting, and encourage the patient to seek treatment elsewhere, at another public hospital. This information and guidance must be updated and reinforced upon each visit.

Patients would thus be made aware of the need to look for another service, to try to have their surgery scheduled for the near future. If the patient chooses to do otherwise, this will be his own free and educated decision – something totally different from the previously proposed scenario. Additionally, the health professional's anxiety is also mitigated as he knows that the issue is being approached in a serious and consistent way. The patient no longer represents an unsolvable problem – a situation that oftentimes impacts the quality of the service rendered. The physician is restituted to his health-promoting role, limited as he is by the conditions of the health institution he works for.

Another benefit of having a line that is more representative of the real problem is that the doctor is better equipped to submit the issue to the appreciation of the authorities and to fight to increase the number of surgical procedures offered.

The only reasonable justification – as controversial as it is— to denying service to patients is not the waiting time for surgery but the fact that outpatient clinics are overcrowded. It can be argued that accepting a new patient has a direct negative impact on the service that can be provided to another patient. This provides for a more complicated issue from the ethical point of view, one that involves concepts such as priority and severity, and that are discussed below.

### “Wait at home. We will call you when we have an opening”.

Another common practice that should be avoided at all costs is to register patients and send them home until there is a surgery opportunity. Again, this attitude usually hides an anxious doctor who does not want to see the patient lest he be reminded of the fact that he is not able to “solve the patient's problem” —for he believes that, except for surgery, there is nothing that he can do to mitigate the problem.

However, alleviating the physician's anxiety is directly proportional to a growing anxiety on the patient side. Without follow-up visits, patients focus all their hopes on the expected call, generating profound psychological impact and unnecessary stress.

This attitude also serves to distort the reality of medical treatment in our specialty, reducing everything to the surgical procedure itself. Again, it must be highlighted that even though the surgery maybe essential to the treatment of a given patient, it is seldom the final solution to the problem. We know, for example, that although surgery is mandatory for cholesteatoma or for extensive nasosinusal polyposis, it will hardly be a final and absolute treatment for these conditions. Both diseases have a high relapse rate and may require several interventions. Secretions may never stop in the cholesteatomatous ear, and hearing may even worsen after surgery. How, then, can we justify to the patient that he is being “followed up” at a distance, and make him place all bets on the rescuing power of the surgical procedure ahead of him, if symptoms may persist, or even worsen, after two or three interventions? This would make for a disastrous relationship. The physician will probably put the blame on the health system or on the conditions inherent to the disease, oblivious to the fact that if this very patient were to be periodically seen and treated during acute presentations of the disease, being made aware of the condition as it evolves, he would definitely develop a different relationship with the doctor and with the disease.

Therefore, patients waiting for surgery should follow a schedule of periodic appointments, according to the disease and the conditions provided by the health facilities. During these visits, in addition to clinical treatment, the guidelines previously mentioned should be reinforced, register data should be updated and the patient should be informed of the line status.

### Sizing the problem to find a solution

No feasible, impacting solutions can be found if the issue of waiting times is not adequately dimensioned. The first step to find realistic solutions is to have a good grasp of the real size of the problem. Otorhinolaryngology services must periodically record and update information on the several waiting lines. It is necessary to have an accurate idea of the number of people waiting for a given kind of surgery, the number of serious cases and the morbidity/mortality rates at the waiting lines before we can make demands for more surgery facilities or more professionals to the hospital management or to the competent authorities^3^. Accurately presented information adds to demands legitimacy, increasing the chances of positive responses. During the last years, there have been many cases of extra funding granted, at the state and federal levels, to promote elective otorhinolaryngology surgery joint efforts based on well-structured endeavors.

The easiest time to measure, as we have already said, is the last phase; the one that goes from the moment the patient is included in the list until surgery performance. In order to obtain such information, the date the patient was admitted in the waiting line must be indicated in the patient's record. This will allow us to assess average and maximum times in waiting. These data are actually more important than the number of patients waiting for a specific surgery.

In England, the number of patients awaiting otorhinolaryngology surgery throughout the country has been reasonably constant for the last 50 years —varying between 100 and 150 thousand people^4.^ The average waiting time, however, has dropped significantly since the beginning of the 90's. In 1989, there were around 25,000 people waiting for more than one year for some type of otorhinolaryngologic surgery, whereas in 1996 this number was only 532 patients for the entire country.

As we have said, it is not enough to have an adequate and updated record of patients waiting for surgery. At some point during treatment the patient must also be asked to inform the time span from the beginning of the disease until the surgical procedure^5^. This is unquestionably more complex an issue. In our service, we choose to fill out a form *after* the surgery, at the moment the patient is discharged from hospital. We also ask patients how the visit at the otorhinolaryngology clinic was obtained.

### Transparent information

The mere maintenance of a formal and transparent record of waiting lines serves to prevent undue tampering with the lists. Wide dissemination of lists among the physicians working for the service operates as a true safety system —a virtual surveillance camera—, making it much more difficult to engage in practices such as mysteriously placing patients at privileged positions in the list. Lack of organization is always a strong ally to injustice.

Each waiting list should be assigned to the responsibility of a physician who is to be forwarded all the information regarding patients operated on, patients removed from the list or changes in patients' data; preferably on a weekly basis. The updated list must then be printed out and distributed to the physicians in the service, or be made otherwise available, at the secretary of the facility, for example. Thus described, this may seem a procedure demanding a lot of work. However, if this list is kept in an electronic file in a text editor or in a spreadsheet, updating will take only a few minutes, and this will constitute a low-cost, high-value routine for the health service.

Likewise, patients must not be denied access to this information. They should be told, as often as possible, how many patients lie ahead, waiting for surgery, and why. Instead of making for disgruntled patients, conveying this information usually makes them comfortable and less anxious, for they know that their case is being followed up in a serious and organized fashion.

### Data Validation

Surgery is not the only way out from a waiting list. Other four possibilities must be monitored by the service in order to keep the list updated. Patients may die, move to another town, undergo surgery at another facility or no longer require surgery (surgical indication reversal).

If visits are regularly repeated, it is easy to maintain an updated list and to notice the absence of any patient (in which case an attempt should be made to contact this person). In 1990, a study conducted in England showed that mere validation of the waiting list by detecting double entries or patients that should be removed from the list due to the reasons mentioned above resulted in a 44% reduction of the list (3,531 patients, from 8,004 originally). In 2004, a single federal hospital in Rio de Janeiro (National Institute of Traumatology-Orthopedics) reduced the names in waiting lists by 2,000 entries just by having the patients' records revalidated^6^.

### Resorting to the “wait and see list”

Many cases in otorhinolaryngology demand time and patient following up before an indication for surgery can be made. Tonsil and adenoidal surgeries are particularly good examples along these lines. Many of the children on the waiting list outgrow their condition as they wait for their turn to be operated on, to the point that some of them no longer need to be submitted to the procedure. Other cases may be clinically monitored for a while before we opt for surgery. This practice may lead to injustices, as patients that have been followed up for many years have to be placed at the end of the line when we choose to operate a condition which was actually present at the very first visit.

For these cases, it may be useful to have a “wait-and-see” list, to include patients whose indication for surgery is still unclear. This would allow the possibility of transferring patients from one waiting list to another without adding to the total waiting time.

### Service priority

Surgical service priority reflects the order in which patients will be submitted to surgical procedure. So far, time in the waiting list has been the chief priority criterion. However, this is clearly not the only factor do be considered. Patients in need of emergency surgeries (acute mastoiditis or sinusitis with intra-orbital complications) are extreme examples of cases that should be given priority regardless of waiting times.

Likewise, prioritization should also apply to elective surgeries, according to the severity and urgency of each case. Patients with severe presentations must be operated on before those with less severe cases, regardless of the time they have been registered in the service. However, such priority criteria must be clearly and well established to ensure smooth service running.

When assessing severity, one should consider extent of suffering, limits to activities or risk of death imposed by the disease. For urgency criteria, consideration must be given to severity and possible benefits of surgery *vis-à -vis* the disease's natural history, in addition to social and philosophical factors^7^. A case of laryngeal cancer at its inception, for example, is not a serious case at the moment, since mild dysphonia is the only presentation, but it is an urgent case, for surgery has a strong impact in disease evolution. On the other hand, although severe, a terminal patient is not an urgent case, as surgery has less impact on the natural history of the disease. Based on these concepts, each facility should set its own priority criteria for each surgery.

Generally speaking, some criteria must be highlighted:

1. Complications history
  a. Systemic Complications
  b. Complications in adjacent organs and structures.
  c. Local complications.
1. Patients with serious comorbidities
2. Patients with clinical or radiological signs of advanced disease.
3. Patients under 18 and the elderly.
4. Socioeconomic factors.

The medical history of complications from diseases —infectious or not—, may be the most important factor to take into consideration as it represents an increased risk of death. Complications must be classified according to severity and relapse likelihood. As a rule, intracranial infections (meningitis, intracranial abscesses, empyema) are deemed the most serious complications due to the risk of death and high relapse rates intrinsic to these conditions. Next we have complications in adjacent organs (as orbital complications in nasal polyposis) and those restricted to the disease's target organ (such as facial paralysis in cholesteatoma).

Patients with serious comorbidities are another priority group. Comorbidities are construed as other systemic affections –whether related or not to the otorhinolaryngologic disease— that contribute to a more serious scenario due to the possibility of complications ensuing from the mutual interaction of the two diseases. Classical examples are the association between polyposis and asthma, or tonsil and adenoidal hypertrophy and sleep obstructive apnea syndrome. However, there are other systemic affections which, even if not directly related to the otorhinolaryngologic disease, must be taken into consideration: kidney failure, transplant patients (or transplant candidates), AIDS patients, severe diabetics, etc.

Patients with clinical or radiological evidence of advanced disease, suggesting increased possibility of evolving towards complications, must also be given priority.

Cholesteatomas with advanced erosion of the *tegmen timpani* are included in this group.

According to the “child and adolescent statute” and the “elderly statute” —both already in force in Brazil—, these age bracket groups must always be given priority in any public hospital services as well as in the implementation of any health policies. Therefore, they are also considered as priority cases.

Considerations of socioeconomic nature, although important, are very controversial when it comes to setting up priority criteria. Some services prioritize surgeries on people who live in distant municipalities or even other states, understanding that long periods of waiting away from home is disruptive and adds to the patient's suffering. Other institutions may deem relevant to consider issues of a more subtle nature, such as the disease's impact on the patient's professional life; or choose to prioritize patients who care for sick relatives and have no time to take care of their own health. The possibilities are endless. Thus, it is necessary to have an open attitude, discussing the criteria that should be taken into consideration as well as specific cases. The surgical team's free understanding and common sense cannot be left aside, as long as the same criteria are applied to all cases.

### Changes in health policy impacting the waiting lines

So far we have discussed several measures and concepts for local level implementation that would have great impact on surgery waiting lines in our specialty. However, it would be naïve not to consider the fact that deep changes in health policy are required so that the problem may be brought under control once and for all.

Although an in-depth discussion of Brazilian health policy is not within the scope of this article, we deem relevant to make a few comments on specific issues with heavy impact on the problem of waiting lines.

### Salary x Fee-for-service Compensation

The OECD (Organization for Economic Co-operation and Development), an institution created more than 40 years ago by first-world countries, conducted a study where member countries were divided into two groups: those where waiting lines for surgery were a problem (Australia, Canada, Finland, Ireland, Italy, The Netherlands, New Zealand, Norway, Spain and England); and those countries where waiting times were not significant (Austria, Belgium, France, Germany, Japan, Luxemburg and Switzerland). The study compared some aspects of health policy in both groups^10^.

The first conclusions were already expected. Countries which invest more in hospital beds, physicians, surgery rooms and technology had fewer problems with waiting lines for surgery. The multivariate statistical analysis showed that each one of these items were individually significant.

However, other conclusions reached by the study were surprising. According to the survey, public service physicians' compensation basis, analyzed in isolation, had a strong impact on waiting times for surgery. Countries that adopt a per-service-fee or mixed systems (salary plus productivity) presented less of a problem in terms of waiting lines than those countries where physicians earn fixed salaries.

This finding is supported by several other studies. A recent review of the compensation methods adopted for physicians working with primary health care adopted strict inclusion criteria, valuing randomized trials, and concluded that payment per procedure results in better quality service than the waged method^11^. As far as surgery is concerned, a randomized study conducted by Siu et al. in the United States showed that the rate of elective surgeries is greater in hospitals where surgeons are paid per procedure than in those where salaries were the form of compensation. The rate of emergency surgeries was found to be the same in both systems. Two other papers comparing American hospitals with different compensation basis arrived at the same conclusion13., 14.. Ransom et al., in another study design, noticed a 15% drop in the number of elective surgeries in a hospital where physicians remuneration changed from fee-per-service to salaries^15^.

Payment per surgery seems to be the most efficient and satisfactory form of compensation for surgeons, anesthesiologists, hospitals and for the patient.

### Joint efforts, Campaigns and Resources from the Compensation and Strategic Actions

Whenever the issue of long waiting lines comes up, the idea of joint effort surgeries comes to mind. By joint effort we mean a concentrated effort of surgical services in order to carry out a larger number of specific surgeries within a short period of time. In order for the joint effort not to have a negative impact on service smooth running (outpatient clinics, examinations, etc.) nor on other elective surgeries of the same specialty this endeavor must rely on extra funds, whether from the hospital budget itself or from additional local, state or federal spheres. Joint efforts are actually very valuable as emergency procedure, chiefly when it is regarded as an initial measure for the implementation of a new health policy within the specialty.

In 1999, the Ministry of Health created the National Campaign for Elective Surgeries, aiming at organizing and funding joint efforts for surgeries^16^. Problems chosen for surgery were those considered “strategic” and mainly those more relevant and with longer waiting lines in public services. Several procedures were included in the project, such as cataract, inguinal hernia, prostate and varicose veins. In April 2001, a new ordinance determined that high-complexity actions and “strategic actions” were to be funded by FAEC (Fundo de Açõ es Estratégicas e Compensaçã o – Strategic Actions and Compensation Fund)^17^. From thereon, several joint efforts were implemented with FAEC's resources, whether or not directly connected to the campaign, as well as other surgeries of various specialties, including otorhinolaryngologic surgeries all over the country.

Notwithstanding, in the last five years the time period for these campaigns has been expanded by successive government ordinances. In spite of an almost three-fold increase in the number of elective surgeries, adoption of these campaigns in a permanent fashion has generated undesirable distortions.

Joint efforts and strategic programs originally meant as emergency actions became the *modus operandi*, a means for ensuring the survival of surgical specialties, a mechanism to obtain “extra-ceiling funds”. This extended policy has created a race for strategic programs accreditation, now treated as chickens that lay golden-eggs, the source of revenues which, oftentimes, amount to more than the original budgets of the institutions. This creates a scenario of unfair compensation for certain specializations and procedures that cannot be deemed “strategic” and, therefore, cannot be granted FAEC's extra funds. This classification is clearly influenced by lobbies and political interests.

We need to fight for these programs to be gradually transformed into health policies with a more wide-encompassing action, allowing for a more equitable distribution of health funds and, therefore, a more equitable compensation of surgeries by SUS. The implementation of our current health policy for hearing is a true step along these lines^19^ that should serve as a starting point for an even more complete action covering all of otorhinolaryngology and ensuring the much desired universal access.
